# Explaining the mechanisms behind niche dimensionality and light-driving species diversity based on functional traits

**DOI:** 10.1038/s44185-024-00049-3

**Published:** 2024-07-25

**Authors:** Zhengwei Ren, Wei Zhao, Ning Chen, Xiaolong Zhou

**Affiliations:** 1 College of Ecology, Lanzhou University, No. 222 Tianshui South Road, Lanzhou, 730000 Gansu China; 2 College of Ecology and Environment, Key Laboratory of Oasis Ecology, Ministry of Education, Xinjiang University, Urumqi, Xinjiang 830046 China

**Keywords:** Ecology, Ecology

## Abstract

Two prevalent ecological mechanisms, niche dimensionality and light asymmetry, may well explain species loss with fertilization gradients in grassland communities. Although there is still controversy surrounding the two competitive mechanisms that maintain species coexistence, few studies have examined the patterns of change in dissimilarity in species composition (β-diversity) and the relative explanatory contributions of plant functional traits to α- and β-diversity when multiple resources are added. To clarify this knowledge gap, we conducted a 6-year experiment of resource addition in an alpine meadow on the Qinghai-Tibet Plateau to assess how species richness and spatial β-diversity are affected by increasing numbers of added resources (NAR) and light limitation. Our results found that both NAR and light limitation led to decreased species richness, suggesting that niche dimensionality and light asymmetry may contribute equally to species loss, rather than either alone. Moreover, NAR is the primary factor responsible for the increase in β-diversity, which exhibits a negative relationship with species richness. Furthermore, the increase in height is the most likely explanation for β-diversity, while the increase in SLA is the most likely explanation for species richness, thereby indicating the changes in species richness and composition can be effectively explained by the response of certain morphological functional traits with the addition of multiple resources. Future research should focus on the complex interactions of different ecological mechanisms that contribute to the maintenance of biodiversity in grassland ecosystems all over the world.

## Introduction

Continuous fertilizer application and atmospheric deposition pose significant risks to the number of plant species, community composition, and ecosystem functioning in terrestrial ecosystems^1–4^. In grassland communities, a negative correlation between species diversity and fertilization gradients has been extensively studied^5–7^. Various hypotheses have been put forward to explain this phenomenon and to determine the underlying competitive mechanisms responsible for species loss^8–11^. However, the causal links between species diversity and competition for resources are still a matter of controversy^12^.

To date, a large number of experimental studies have demonstrated that species loss due to fertilization can be explained by either competition for light or niche dimensionality^10–13^. In particular, studies based on the light competition hypothesis since the 1970s have suggested that fertilization-induced increased biomass (primarily grass) leads to a reduction in light availability, which accelerates the competitive exclusion process via size-asymmetric light competition^14–16^. In addition to aboveground competition, competition for belowground resources can also result in species loss^17,18^. For instance, niche dimensionality hypothesis offers an alternative mechanistic explanation, suggesting that the reduction in limiting resource numbers (as opposed to number of added resources [NAR]) causes a decrease in the number of species that can coexist within the community^17^. In recent decades, various grassland studies, including many local^19–21^, global^18^, and meta-analyses^12^, have examined the drivers of species loss with the addition of multiple resources. Specifically, Harpole et al. and DeMalach and Kadmon have recently debated the relative importance of light competition and niche dimensionality in structuring grassland species diversity by analyzing the same dataset from a global experiment (45 sites from five continents)^10,11^, and assess the importance of any one mechanism. However, a significant limitation of the debate was that it did not attempt to test the equal roles of two non-exclusive hypotheses. This was because each of the two hypotheses could lead to biased conclusions regarding the effects of the underlying mechanisms^22^.

Functional traits offer a valuable perspective in unraveling the complex interaction of multiple impacts on species richness, community composition and functioning in natural ecosystem^23,24^. According to the functional-based hypothesis, fertilization will promote species possessing resource-acquisitive traits (e.g., tall stature, high-specific leaf area [SLA]), which are advantageous under resource-rich conditions, to the exclusion of other species possessing resource-conserving traits (e.g., short stature, low SLA)^25^. In many cases, functional traits, such as height or SLA, are often closely correlated with competition for light, with a switch to aboveground competition occurring primarily when multiple resources are added but shading is intense^26,27^, contributing to understanding divergent trait responses to fertilization among species^28^.

Although α-diversity (species richness) has received considerable attention in its response to niche dimensionality^17,18^, there is a notable knowledge gap regarding the important role and changes patterns in β-diversity. As is well known, β-diversity provides insights into the mechanisms driving species compositional changes and their consequences for ecosystem functioning^29^. In contrast to the study examining the response of species richness to fertilization^13,29^, other fertilization studies, from the other side, assess whether the change in β-diversity during community assembly processes is correlated with variations in dispersal or niche-selection associated traits^29,30^. This is because functional strategy differentiation among species drives the maintenance^31^ and distribution^32^ of species. Classical resource competition theory predicts that species have specific trade-offs for different limiting responses. Consequently, altered resource supply ratios are expected to influence β-diversity via their effects on competitive relations^27^. For instance, following fertilization, species competition for soil and light resources could result in the dominance of grass species at the expense of numerous small forbs species^32^, thereby homogenizing the structure of grassland communities (i.e., increasing β-diversity). Consequently, the change pattern in β-diversity can also reflect the replacement of species with traits of low growth rate and low height to species with traits of high growth rate and greater height. This supports the robust explanatory ability of functional traits on β-diversity, as evidenced by previous studies^27,30,33,34^. The objective of this study is to investigate the pattern of change and underlying mechanisms of β-diversity in relation to niche dimensionality, with a focus on functional traits, beyond assessing the potential role of functional traits explaining species richness loss. This will provide valuable insights into the relationship between limiting resources and α- and β-diversity.

In this study, we conducted a long-term study on grassland resource addition at the Gansu Gannan Grassland Ecosystem National Observation and Research Station in an alpine meadow community of the eastern Qinghai-Tibet Plateau, Lanzhou University, China. The study aimed to address the following questions. First, we analyzed the changes in species richness, biomass, and photosynthetically active radiation (PAR) along NAR and their ecological relationships, and then used the first type of structural equation model (SEM) to assess the equal roles of niche dimensionality and light asymmetry as drivers of species loss (α-diversity). Specifically, we incorporated the effects of NAR, biomass, and PAR into an SEM. We included both direct and indirect pathways in our proposed hypotheses, and hypothesized that the reduction in species richness can equally be driven by both NAR and light asymmetry, instead of only one of them. Second, beyond α-diversity, analyzing the relationship between both β- diversity and community-level weighted means (CWM) of traits (SLA and height). Third, after considering the private relationship between functional traits, species richness and β-diversity, we applied a second type of SEM to discriminate predictor ability of functional traits (height and SLA) on species richness and β-diversity with resource addition.

## Results

### The driving roles of niche dimensionality and light competition

**Fig. 1:**
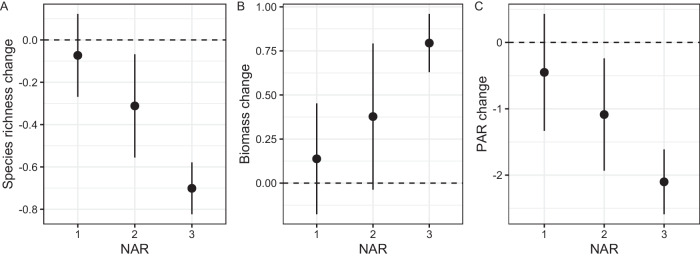
Changes in species richness, biomass and light. From control plots, increasing NAR led to a decrease in species richness per year (**A**), increase in biomass per year (**B**), and decrease in the proportion of PAR reaching the ground surface per year (**C**). Dashed line indicates control plots (zero value). NAR number of added resources, PAR photosynthetically active radiation.

**Fig. 2:**
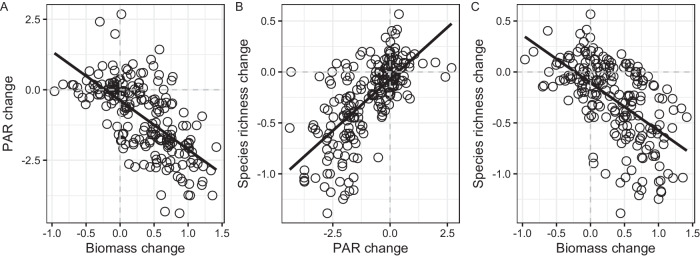
Relationship between species richness, biomass, and light. **A** The proportion of PAR reaching the ground surface decreased with increasing biomass. **B** Species richness increased with increasing proportion of PAR reaching the ground surface. **C** Species richness decreased with the biomass increased. Dashed line indicates control plots (zero value) and black circle represents the log_10_-transfromed change ratios across each year. PAR photosynthetically active radiation.

In terms of change rate, we found that species richness decreased with NAR in the experiment plots compared to control plots (Fig. 1A; *F* = 16.64, *p* < 0.0001). Conversely, biomass increased with NAR (Fig. 1B; *F* = 6.77, *p* = 0.0012), while PAR reaching the soil surface reduced with NAR (Fig. 1C; *F* = 8.56, *p* = 0.0003). In terms of the contribution of functional group biomass to community biomass, grass (*R*^2^ = 0.45, *p* < 0.0001) and sedge (*R*^2^ = 0.14, p < 0.0001) biomass accounted for a large proportion of the increased community biomass, whereas forb (*R*^2^ = 0.15, *p* < 0.0001) and legume (*R*^2^ = 0.05, *p* = 0.0014) biomass did not (Supplementary Fig. 1). Furthermore, PAR decreased as biomass increased (R^2^ = 0.34, *p* < 0.0001; Fig. 2A). In contrast, species richness increased as PAR increased (*R*^2^ = 0.45, *p* < 0.0001; Fig. 2B), and a negative relationship was found between species richness and biomass (*R*^2^ = 0.34, *p* < 0.001; Fig. 2C).

**Fig. 3:**
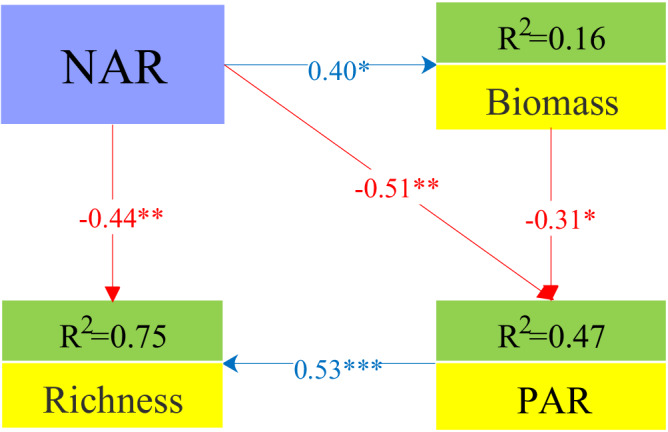
Structural equation model examining the effect of NAR and light on species richness in the last experimental year. The *R*^2^ values in the boxes indicate the proportion of variance explained by the model for each response variable. Estimates shown are standardized path coefficients. The arrows reflect the relationship of positive (blue) and negative (red) paths. Significance levels of path coefficients are shown as * (*p* < 0.05), ** (*p* < 0.01), and *** (*p* < 0.001). Global model: Fisher’s *C* = 2.414, degrees of freedom (d.f.) = 2, Akaike Information Criterion (AIC) = 293.76, *p* value = 0.299. NAR number of added resources, PAR photosynthetically active radiation.

When assessing the significant role of NAR and light asymmetry driving species loss, the first SEM found that NAR significantly increased biomass, which led to a significant decrease in PAR reaching the ground surface, and in turn reduced PAR reaching the ground surface, indirectly decreasing species richness. Meanwhile, NAR had a direct negative effect on species richness. These results showed that NAR and light limitation equally lead to diversity loss through direct or indirect pathways (Fig. 3, Supplementary Table 1).

### Change in functional traits and their correlation with species diversity

**Fig. 4:**
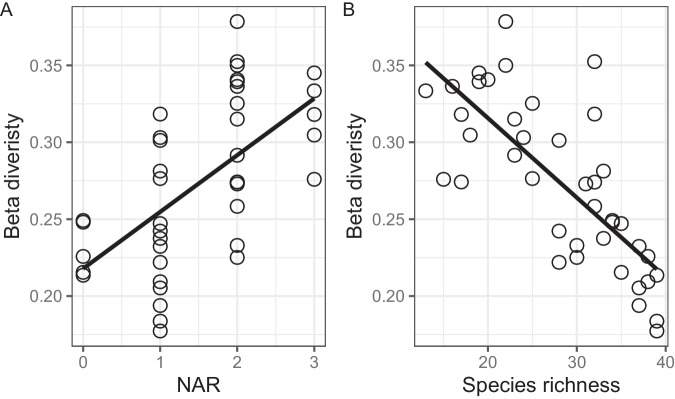
The change in β-diversity with increasing NAR and its relationship with species richness in the last experimental year. **A** β-diversity increased significantly with the NAR. **B** β-diversity decreased significantly with species richness. *NAR* number of added resources.

**Fig. 5:**
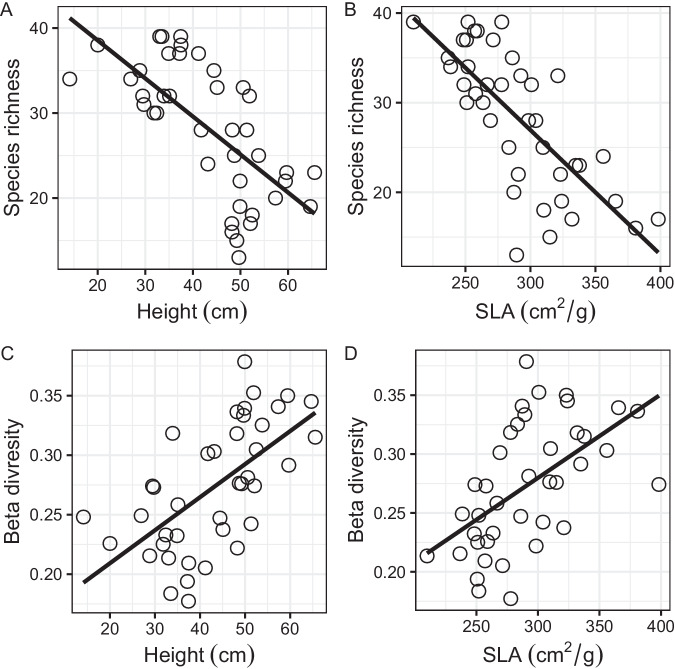
Prediction of the effect of functional traits on species richness and β-diversity in the last experimental year. **A**, **B** Species richness decreased with increasing height and SLA; **C**, **D** β-diversity increased with increasing height and SLA. SLA-specific leaf area.

NAR resulted in a significant increase in height (*F* = 23.73, *p* < 0.0001) and SLA (*F* = 13.00, *p* = 0.0009) compared to the control plots (Supplementary Fig. 2), indicating a consistent response of these two traits to NAR. PAR decreased with increasing height (*R*^2^ = 0.58, *p* < 0.0001) and SLA (*R*^2^ = 0.47, *p* < 0.0001) (Supplementary Fig. 3). Additionally, β-diversity increased significantly with NAR (*R*^2^ = 0.34, *p* < 0.0001) and had a significant negative relationship with species richness (*R*^2^ = 0.56, *p* < 0.0001) (Fig. 4A, B). Moreover, species richness decreased with increasing height (*R*^2^ = 0.45, *p* < 0.0001) and SLA (*R*^2^ = 0.54, *p* < 0.0001), suggesting that functional traits induced by light competition played an important role in reducing species richness (Fig. 5A, B). Furthermore, the dissimilarity of community composition was influenced by functional traits, as evidenced by the increase in β-diversity with height (*R*^2^ = 0.30, *p* < 0.001) and SLA (*R*^2^ = 0.38, *p* < 0.0001) (Fig. 5C, D).

### Explaining changes in species diversity based on functional traits

**Fig. 6:**
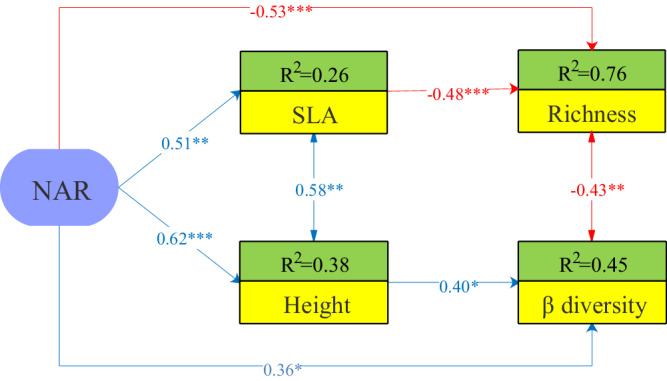
Structural equation model investigating the effect of NAR and functional traits on species richness and β-diversity using data from the last experimental year. The *R*^2^ values in the boxes indicate the proportion of variance explained by the model for each response variable. Estimates shown are standardized path coefficients. The arrows reflect the relationship between positive (blue) and negative (red) paths. Significance levels of path coefficients are indicated as * (*p* < 0.05), ** (*p* < 0.01), *** (*p* < 0.001). Global model: Fisher’s *C* = 3.922, Akaike Information Criterion (AIC) = 395.12, degrees of freedom (d.f.) = 4, *p* value = 0.417. NAR number of added resources. SLA specific leaf area.

The second SEM result found that NAR had a direct and important driving influence on the reduction in species richness and the increase in β-diversity. Moreover, species richness decreased with increasing SLA, while β-diversity increased with increasing height, which implied that the response of different traits under light competition explained the contrasting aspect of species diversity (α- and β-diversity). Furthermore, the results also showed that the negative significant relationship between species richness and β-diversity resulted synergistically from functional traits and NAR (Fig. 6, Supplementary Table. 2).

## Discussion

Numerous experiments manipulating multiple resources have strongly supported niche dimensionality^17,18,20^ or light competition^15,16,35–39^ hypotheses. To further investigate the relationship between species diversity and resource competition, we conducted a factorial experiment involving the addition of multiple resources, which demonstrated the conjoint important role of both niche dimensionality and light competition in driving species loss. Additionally, our study demonstrated that CWM values of height and SLA increases significantly with NAR; importantly, our results show that inconsistent changes in species richness (α-diversity) and community composition (β-diversity) mediated by light availability and NAR are explained by SLA and height, respectively.

The relative importance of niche dimensionality and the light competition in driving species loss in grassland communities has been discussed extensively among ecologists^10–12^. At present, niche dimensionality hypothesis predicts species richness will decrease with NAR if species richness is a function of niche dimensionality, i.e., the number of limiting resources^17^. Thus, our results showed that NAR had a significant negative direct effect on species richness, supporting those of previous studies that explained species loss using NAR as strong evidence for niche dimensionality^17,18,20^. Previous studies using the same dataset measured the effect sizes of NAR, biomass, and PAR on species richness. All variables were included as potential mediators of the effects of fertilization on species richness, suggesting that light competition may better explain species loss than niche dimensionality^10,12^. However, we do not think so. For example, in our study, the result found that, in addition to NAR, light competition had a significant negative impact on species richness through increased biomass. Specifically, the negative correlation between species richness and biomass suggests that increased biomass-induced light competition is also an important mechanism for species loss. We posit that biomass does not typically impact component species evenly when responding to resource addition^40^. Therefore, the reduction in species richness caused by light limitation likely arises from changes in the biomass of different species or specific functional groups^38^. In our study, grass biomass significantly contributed to community biomass in comparison to other functional groups like sedges, forbs, and legumes due to its competitive advantage in exploiting soil nutrients and fast growth rate. Additionally, we observed that *Elymus nutans* accounted for a large proportion of the increased grass biomass and was the dominant species in fertilized plots in a previous study^19,32^. We therefore believe that size-asymmetric light competition may play an important role in species loss by increasing size inequalities between grass and non-grass species^32^. Other studies have shown that fertilization should reduce the ecological niche dimensionality of belowground resource trade-offs, increase community biomass, and ultimately shift belowground resource competition to a single aboveground light competition^10,11^. Our findings, in contrast, highlight that species competition for belowground resources (niche dimensionality) and aboveground competition (light limitation) may equally affect species diversity, rather than either of them. For instance, our SEM results (Fig. 3) indicated that the path coefficient for niche dimensionality was −0.77 (direct and indirect pathways), while the path coefficient for light competition was 0.53 (direct pathway). So, we believe that both niche dimensionality and light asymmetry have an approximately equal influence on species richness.

According to resource competition theory^41^, competitive trade-offs among species for limiting resources can result in changes in resource supply ratios that drive the dissimilarity of species composition. Our findings indicate NAR increased β-diversity, and decreased species richness was significantly associated with increased β-diversity when multiple resources were added, suggesting that eutrophication causes dissimilar changes in community composition that are associated with species loss. We believe that species richness and composition, along with their impact on ecosystem functioning, are directly influenced by NAR. This indicates that resource availability, particularly eutrophication, is a crucial environmental factor that determines the relative importance of niche-based selection versus stochastic assembly processes^42^. For instance, in line with Conradi et al. study^42^, our findings show that NAR imposed a deterministic environmental filter, resulting in the homogenization of communities by selecting a few species (e.g., grass) with superior growth competitive ability for soil and light resources, consequently reducing the importance of stochasticity.

Many studies have examined the links between species diversity and resource availability by testing the potential role of functional traits as predictors of species response to fertilization^25–27,43^. Their findings reveal that species functional traits linked to resource acquisition, such as large stature and high SLA, were more likely to exhibit a greater increase in NPK-fertilized plots than in control plots. Similar findings have been documented in other studies, but only in N-only fertilized grasslands^26,27,44^. Our findings indicate that the functional traits possessed by the dominant species primarily affect species loss through competition for soil and light resources. According to the plant economic spectrum^25^, fast-growing, resource-demanding species (such as those with tall stature and high SLA) enhance a plant’s light acquisition capacity, promoting plant community biomass through fertilization, which can lead to the extinction of slow-growing, resource-conserving species (such as those with short stature and low SLA). Therefore, functional traits are likely to be important determinants of species sensitivity to changes in resource availability^26^. The findings are consistent with those of previous studies linking tall stature and high SLA to resource-rich conditions, as the roles of the two functional traits in determining community assembly processes were different^28^. The SEM results, presented in Fig. 6, demonstrate that NAR contributes indirectly to the increase in β-diversity by promoting height. On the other hand, NAR indirectly leads to a decrease in species richness by increasing SLA. Consistent with previous studies, fertilization usually leads to the dominance of fast-growing and resource-exploitative grass species^27,45–48^. This dominance results in an increase in community-level height due to the presence of tall-statured grass species. As a result, there is a shift in community composition from a co-dominance of grass, sedge, and forb species to the dominance of only a few grass species, leading to an increase in β-diversity. Furthermore, grass species tend to have thinner, denser, and lower SLA^49^, which can lead to a decrease in community-level SLA when they become dominant. However, our study found that increasing NAR increased SLA. One possible explanation for this result is that the response of SLA differs between tall and short species^28^. Recent research conducted at our study site documented an increase in SLA for understory short forb species following nitrogen fertilization^28^. The study suggests that the forb species shifted to a fast-growth strategy to promote coexistence among species. As a consequence, the increase in community-level SLA caused by the increased SLA of understory forb species outweighed the decrease caused by the dominant canopy grass species. These findings highlight that differentiation in functional strategy among species will drive the reduction in species richness and the increase in β-diversity^28^.

Our study needs a more comprehensive mechanistic explanation of how multiple resources affect species diversity. Several other mechanisms were not integrated into our study to elucidate the link between resource competition and species loss. For instance, one hypothesis predicts that increasing soil resources will lead to community self-thinning with increased average individual plant size in a finite unit plot; thus, species richness in finite area plots will be reduced by a pure sampling effect (community self-thinning hypothesis)^50,51^. Another hypothesis predicts that increasing litter biomass production after fertilization may lead to species loss by reducing seedling establishment (litter hypothesis)^52,53^. In contrast, other studies have shown that soil-mediated effects of acidification and depletion of base cations are very important in influencing species richness, even more important than resource competition^12,54^. It is recommended that future studies integrate these various mechanisms into a detailed theoretical framework and models to test the hypotheses^40^. Moreover, although our results suggest that changes in species diversity following multiple resource additions are mainly driven by NAR (i.e., niche dimensionality), we could not consider the identity of resource-specific attributes in our study. For instance, several studies have demonstrated that N or P additions and their interactions could result in diverse effects on species diversity, composition, and biomass in grassland communities^7,40,54^. Future theoretical studies that concentrating on resource-specific variations between N and P may help us to provide more mechanistic explanations with more accurate predictions, such as the possibility that substitutable forms of N and P affect species diversity and other ecological attributes under N and P limitation^40^.

In summary, our findings dissect the controversy between Harpole and DeMalach & Kadmon regarding the roles of NAR and light availability in structuring species diversity, and highlight that niche dimensionality hypothesis and light competition hypothesis can equally explain changes in species diversity including α- and β-diversity. Moreover, our findings demonstrate that specific functional traits can be considered useful generalizable currencies, either for predicting species vulnerability to changing resource availability or for understanding compositional differences between communities. Our study has significant implications for advancing our knowledge of the impacts of eutrophication on the biodiversity and composition of alpine meadow in the Qinghai-Tibet Plateau. Future studies should investigate whether eutrophication induces species loss and compositional changes by multiple mechanisms in large-scale global grassland ecosystems.

## Methods

### Study sites

Our experimental site was located in the Gansu Gannan Grassland Ecosystem National Observation and Research Station of Lanzhou University (Azi site; 33°58’N, 101°53’E, altitude 3500 m) on the eastern Qinghai-Tibet Plateau. The area has a plateau continental climate, characterized by a mean annual temperature of 2.6 °C, with temperatures ranging from −10.2 °C in January to 12.5 °C in July. Over the past decade, the area has experienced a mean annual precipitation of 617.4 mm, primarily during the short and cool summer. The experimental area exhibited homogeneity in community composition and structure, consisting mostly of natural grassland dominated by Poaceae species such as *Elymus nutans*, *Poa poophagorum*, and *Festuca ovina*, as well as Cyperaceae species such as *Kobresia graminifolia* and *Carex kansuensis*. The site had no anthropogenic history, such as plowing, fertilization, fire, or species introductions, for a long period^19,21^. Prior to the study, the experimental site experienced irregular grazing by livestock (i.e., yak) at a rate of 300 yak per hectare annually during the winter season, except for the vegetation growth season between April and mid-September, when the animals were transferred to other pastures.

### Experimental design

The experimental design and protocols reported here follow those used in Ren et al. study^19^. The multiple-resource addition experiment adopted a three-factorial design comprising control plots with no manipulation and additional plots with nitrogen (N), phosphorus (P), and potassium (K) for eight nutrient-addition treatment combinations. These treatments were then scored from 0 to 3 based on the number of resources added. The control plots received a score of 0, while single resource additions (N, P, or K) received a score of 1, double resource additions (NP, NK, or PK) received a score of 2, and triple resource additions (NPK) received a score of 3. Each of the eight treatment combinations (2 × 2 m) was arranged in five replicate blocks, with all plots separated by 1 m buffer zones. Nitrogen, phosphorus, and potassium nutrients were applied to the experimental plots annually, while micronutrients were applied once at the start of the experiment to avoid toxic levels. Nutrient application rates and sources were as follows: 10 g N m^2^ yr^−1^ as ammonium nitrate [NH_4_NO_3_], 10 g P m^2^ yr^−1^ as triple superphosphate [Ca(H_2_PO_4_)_2_], and 10 g K m^2^ yr^−1^ as potassium sulfate [K_2_SO_4_].

### Data collection

Here, we present experimental data spanning from 2007 to 2012. During the peak plant growth season each year (mid-August), we recorded the number of species within a 0.5 × 0.5-m permanent plot, representing species richness. Afterwards, aboveground live biomass was harvested by cutting a 0.5 × 0.5-m strip of vegetation in each plot and sorting it into four functional groups: grasses, sedges, forbs, and legumes. Biomass samples were dried in separate marked paper bags at 75 °C for 48 h and weighed. The photosynthetically active radiation (PAR, μmol photons per m^2^ per s) reaching the soil surface was measured (Z830 SpectraPen Mini, Czech Republic) and the proportion of light transmitted relative to the canopy above was calculated.

The height (cm) and leaf area (SLA [cm^2^/g] per unit dry leaf mass [g]) of the component species present in the experimental plots were measured. These two traits and their covariation have been demonstrated to have a close relationship to resource competition, such as light competition^55^. Trait data from 8 to 15 individuals per component species were collected in the study area using standard collection and handling protocols^56^, randomly during the last experimental year’s summer. A fresh leaf from each individual was weighed before scanning to measure leaf area using ImageJ software^57^, dried at 75 °C for 48 h, and weighed using a Sartorius balance to an accuracy of 10^−4 ^g. Species abundance was used to calculate the community-weighted mean (CWM) values of the two traits in each plot^58^.

### Statistical analysis

Species diversity is evaluated based on species richness (α-diversity) and spatial compositional dissimilarity (β-diversity). Species richness is determined by the total number of plant species present. The β-diversity for each plot was calculated as the mean compositional change (Bray–Curtis dissimilarity index) among replicates of the same treatment (*n* = 5 replicates per treatment, therefore, each plot has four comparisons) in the last experimental year^59^.

Prior to analysis, the change in each factor was calculated as the log ratio of the treatment response divided by the control, log (R_f+_/R_f-_), where R_f+_ is the log_10_-transformed change ratios of species richness, biomass, or proportion of PAR reaching the ground in each resource addition treatment across years and R_f-_ is the log_10_-transformed change ratios of species richness, biomass, or proportion of PAR reaching the ground in the control plots across years (“center-zero effect”)^12,18,60^. A negative effect size indicates a decrease in the response variable due to the resource addition treatment, while a positive effect size indicates an increase in that response variable due to the resource addition treatment^60^. In addition, we calculated the mean ± s.e. (standard errors) of five plots in each treatment plot to determine the annual rates of species richness, biomass, and PAR; then, we estimated the effects of NAR on species richness, biomass, and light using a linear mixed effect model, where NAR was a fixed factor and block was a random factor. We also tested the relationships between these factors after the addition of multiple resources using regression analysis. A similar regression analysis approach was used to calculate the fitting relationship between each functional group (grasses, sedges, forbs, and legumes) and biomass, and to further assess which types of functional groups contribute to biomass, clarifying their association with increased light limitation with resource addition (Fig. S1).

Using the data of last experimental year, ANOVA was performed to determine the effects of NAR on the two traits (height and SLA), and post-hoc comparisons of NAR gradients were performed using Tukey’s honest significant difference test. After the addition of multiple resources, we separately used linear regression to estimate (1) the relationship between two traits (height and SLA) and PAR reaching the ground surface, (2) response of β-diversity to NAR and its correlation with species richness, and (3) predictive power of two traits (height and SLA) for species richness and β-diversity (higher *R*^2^ values represent better predictive power).

To assess the relative role of niche dimensionality and light asymmetry driving species loss, and the explanatory power of functional traits (height and SLA) on species richness and β-diversity with the addition of multiple resources, we utilized the actual data from the last experimental year. After ensuring consistency and rationality in the data through scale transformation^12^, the two SEMs were conducted using the *piecewise*SEM package^61^. The saturated model, which included all potential explanatory paths (see Supplementary Fig. 4A, B), was constructed with NAR as main factors and blocks as random factors using the lmer () function^62,63^. The two SEMs were later modified by removing non-significant paths. The data were then fitted to the model using maximum likelihood estimation. Finally, the model’s adequacy was evaluated using Fisher’s C statistic, Akaike information criterion (AIC), whole-model *P* value (>0.05). A lower Fisher C, AIC, and a higher *P* value (>0.05) indicate a smaller discrepancy between the predicted and observed values^64^.

All data analyses were conducted using the R 4.4.0 statistical software^64^. All models were fitted using the nlme package, and plots of the fitted relationships between variables were generated using the lm () function. β-diversity (dissimilarity of species composition) was calculated using the vegdist () function from the vegan package. CWM values (height and SLA) were calculated using the dbFD () function from the FD package^65^. The two SEMs were conducted using the *piecewise*SEM and the lme4 package, with all the regression models being fitted by lmer () function^64^. Plots were generated using the ggplot2 package.

### Supplementary information

Supplementary Material

## Data Availability

The data supporting the manuscript are available in the Figshare Digital Repository: 10.6084/m9.figshare.22058168.v3.
