# Supplementary material for: Explaining the mechanisms behind niche dimensionality and light-driving species diversity based on functional traits
Source: NPJ Biodivers. 2024 Jul 25;3:17. doi: 10.1038/s44185-024-00049-3 (PMC11332029; doi:10.1038/s44185-024-00049-3)
Supplement: Supplementary file 1 — Supplementary Material [file 44185_2024_49_MOESM1_ESM.pdf]

Supplementary Material

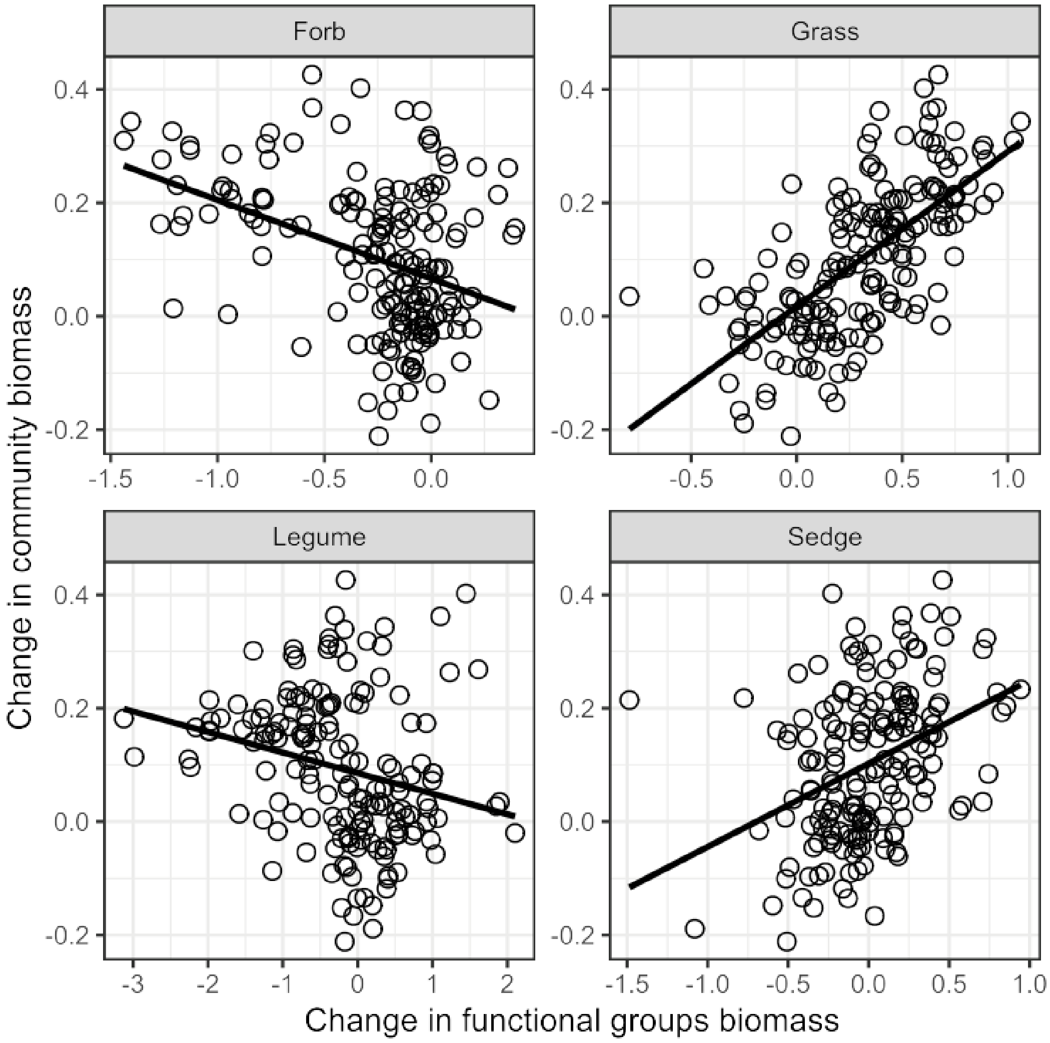

Supplementary Figure 1 Linear regression of community biomass and functional groups biomass. Four functional groups included grass, sedge, forb, and legume. Black circle represents the log_{10}-transfromed change ratio values across each year.

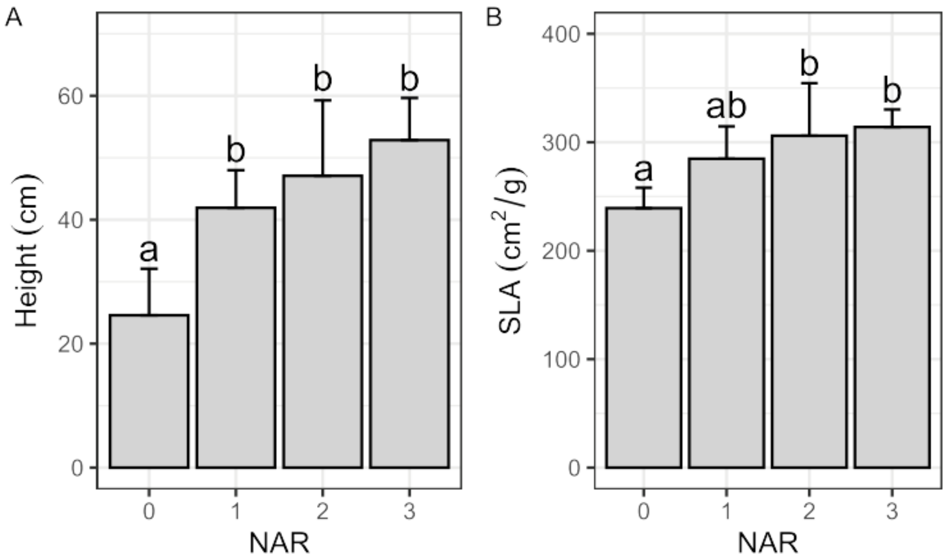

Supplementary Figure 2 The change in community-weighted means of functional traits with increasing NAR in the last experimental year. The significant responses of height (A) and SLA (B) across different NAR gradients. Different letters indicate the level of significance ($p<0.05$). NAR, number of added resources; SLA, specific leaf area.

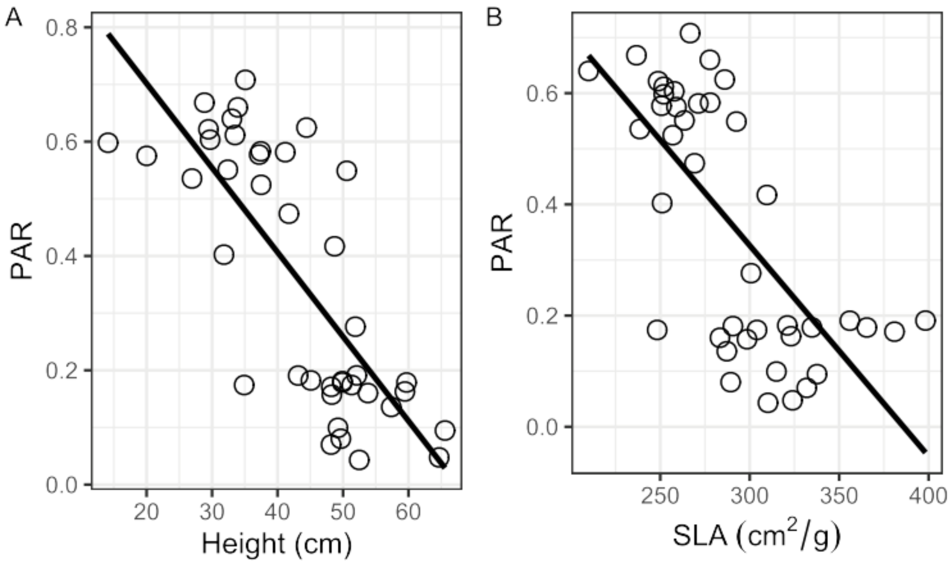

Supplementary Figure 3 Predicting functional traits on light in the last experimental year. The proportion of PAR reaching the ground surface decreased with increasing height (A) and SLA (B). SLA, specific leaf area; PAR, photosynthetically active radiation.

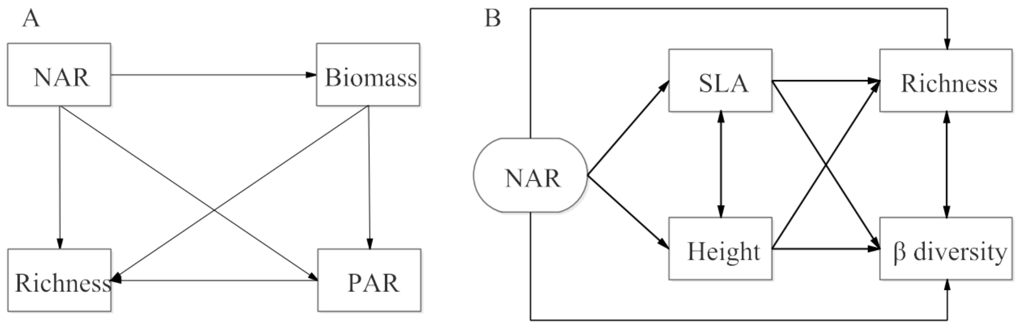

Supplementary Figure 4 The structural equation model of multiple resource addition effects on species diversity. Based on theoretical and empirical predictions, the first type of model (A) tested the relative importance of NAR and PAR reaching the ground surface in driving species richness, and the second type of model (B) tested whether the change in species richness and β-diversity and their relationship could be predicted by specific functional traits (height and SLA) and NAR, considering all plausible pathways. NAR, number of added resources; SLA, specific leaf area.

Supplementary Table 1 Results of the structural equation model of both direct NAR and indirect light limitation with increasing biomass effect on species richness. The table shows the detailed results as shown in Figure 3. NAR, number of added resources. In the Table, given are the unstandardized path coefficients (Estimates), significance values corresponding to the significance-statistic (P-value) and standardized estimate (Std. Estimate). The marginal R^2 and conditional R^2 of the piecewiseSEM anaylsis were shown.

Response variable	Predictor	Estimate	P-value	Std. Estimate
Richness	NAR	-0.500	0.0001	-0.439
Richness	PAR	0.527	0.0000	0.527
PAR	Biomass	−0.309	0.0240	−0.309
PAR	NAR	−0.585	0.0004	−0.513
Biomass	NAR	0.460	0.0103	0.403

Response	Marginal	Conditional
Richness	0.75	0.77
PAR	0.47	0.47
Biomass	0.16	0.16

Supplementary Table 2 Results of structural equation model of NAR and functional traits (height and SLA) impact on species richness, β-diversity, and their relationship. Table shows the detailed results as shown in Figure 6. NAR, number of added resources; SLA, specific leaf area. In the Table, given are the unstandardized path coefficients (Estimates), significance values corresponding to the significance-statistic (P-value) and standardized estimate (Std. Estimate). The marginal R^2 and conditional R^2 of the piecewiseSEM anaylsis were shown.

Response variable	Predictor	Estimate	P-value	Std. Estimate
Beta diversity	Height	0.399	0.0191	0.399
Beta diversity	NAR	0.411	0.0275	0.360
Richness	SLA	−0.482	0.0000	−0.482
Richness	NAR	−0.606	0.0000	−0.532
SLA	NAR	0.576	0.0010	0.505
Height	NAR	0.707	0.0000	0.620
~~SLA	~~Height	0.579	0.0001	0.579
~~Beta diversity	~~Richness	−0.431	0.0031	−0.431

Response	Marginal	Conditional
Beta diversity	0.45	0.46
Richness	0.76	0.78
SLA	0.25	0.25
Height	0.38	0.39
